# An investigation of the factors influencing college students’ rural cultural preservation behaviors in the context of rural cultural revitalization: a hybrid SEM–ANN approach

**DOI:** 10.3389/fpsyg.2025.1716261

**Published:** 2025-12-11

**Authors:** Yuanshuai Yang, Haiqing Liu, Xiaohui Liu, Qian Bao

**Affiliations:** 1 Department of Environmental Design, School of Design and Fine Arts, Qingdao Huanghai University, Qingdao, China; 2 Spatial Design, Graduate School, Hongik University, Seoul, Republic of Korea; 3 Department of Design, Graduate School, Hanyang University, Seoul, Republic of Korea

**Keywords:** rural cultural preservation, behavioral intention (BI), structural equation modeling (SEM), artificial neural network (ANN), social cognitive theory (SCT), theory of planned behavior (TPB)

## Abstract

**Introduction:**

The protection and inheritance of rural culture have emerged as critical components of rural cultural revitalization amid the ongoing implementation of the rural revitalization strategy. College students, characterized by both academic knowledge and social responsibility, have not yet been systematically examined in terms of the mechanisms underlying their participation in rural cultural preservation. Although rural cultural protection has received significant scholarly attention, theoretical and empirical investigations specifically focusing on college students’ behavioral intentions and their determinants remain scarce. An integrated model is proposed based on the theory of planned behavior (TPB) and social cognitive theory (SCT) to examine the effects of attitude toward the behavior (AT), subjective norm (SN), perceived behavioral control (PB), and self-efficacy (SE) on behavioral intention. In addition, the study explores how social support (SS), as an external social contextual factor, indirectly affects behavioral intention by influencing other psychological variables.

**Methods:**

A two-stage analytical strategy combining structural equation modeling (SEM) with artificial neural networks (ANN), was adopted to capture both linear and nonlinear relationships, thereby enhancing the model’s explanatory power and predictive accuracy. A total of 441 valid questionnaires were collected from university students across various regions in China.

**Results:**

The ANN results significantly validated the SEM findings, further reinforcing the reliability of the research outcomes.

**Discussion:**

This study’s innovation lies in the integration of TPB and SCT into a unified framework for modeling the factors that influence college students’ intentions to engage in rural cultural preservation. In addition, the incorporation of a SEM–ANN hybrid modeling technique effectively addresses the limitations of traditional linear models in capturing complex interaction effects. In summary, this study not only broadens the application scope of TPB and SCT in rural cultural preservation research but also provides theoretical foundations and practical implications for the development of educational interventions to enhance college student engagement.

## 1 Introduction

Rural culture has been recognized as the soul and foundation of rural society (Li and Lin, 2022), encompassing rich historical knowledge, folk customs, social values, and practical wisdom (Cheng et al., 2024; Kang, 2023). According to UNESCO (2005), cultural diversity must be protected and transmitted, with rural culture identified as an essential component thereof (Li et al., 2024). In the Chinese context, the protection of rural culture plays a vital role in promoting rural cultural revitalization (Wu and Guo, 2023). However, urbanization and industrialization have significantly impacted rural cultural ecosystems, triggering a preservation crisis (Yang et al., 2021; Guo and Zhang, 2013). Additional challenges include the decline of traditional folk practices, the deterioration of rural architecture, and the generational disconnect among cultural inheritors (Cheng and Zhi, 2024).

College students, as disseminators of knowledge and culture, possess advanced theoretical knowledge, practical expertise, and creative thinking, positioning them as key actors in cultural preservation efforts (China Social Work, 2022; Guangming Daily, 2024; Sohu News, n.d.). As of 2023, over 47.63 million students were enrolled in higher education institutions in China (Ministry of Education Development Planning Department, 2024), providing a substantial human resource base. Thus, empowering students to participate in rural cultural revitalization is not only a timely opportunity but also a social responsibility (Wang et al., 2021; Zhang, 2025). Investigating the factors influencing students’ behavior in this domain is critical to activating their engagement and advancing cultural revitalization initiatives.

Research on the rural cultural protection behavior of college students remains limited, particularly in terms of theoretical modeling. Most existing research has concentrated on practical service models (Zhou, 2024; Wang, 2021), while little attention has been paid to students’ internal behavioral intentions. Moreover, prior studies have primarily relied on traditional linear models, overlooking nonlinear dynamics that reflect the interplay between internal motivation and external social norms—often core components of actual decision-making processes (Cai et al., 2025). Therefore, this study employs structural equation modeling (SEM) in conjunction with artificial neural networks (ANN) to explore the complex interactions between theoretical constructs from social cognitive theory (SCT) and the theory of planned behavior (TPB).

This study aims to systematically identify the key factors influencing college students’ intentions to participate in rural cultural preservation, while achieving dual innovations in both theoretical and methodological dimensions. Specifically, the study: (1) examines the internal psychological mechanisms and external social factors shaping student behavioral intentions; (2) applies a hybrid SEM–ANN framework to model both linear and nonlinear relationships, overcoming the limitations of traditional modeling approaches; and (3) integrates SCT and TPB into a unified analytical framework to improve explanatory power and predictive validity in the context of rural cultural engagement.

## 2 Theoretical framework, research hypotheses, and conceptual model

### 2.1 Social cognitive theory (SCT) and the theory of planned behavior (TPB)

**Figure 1:**
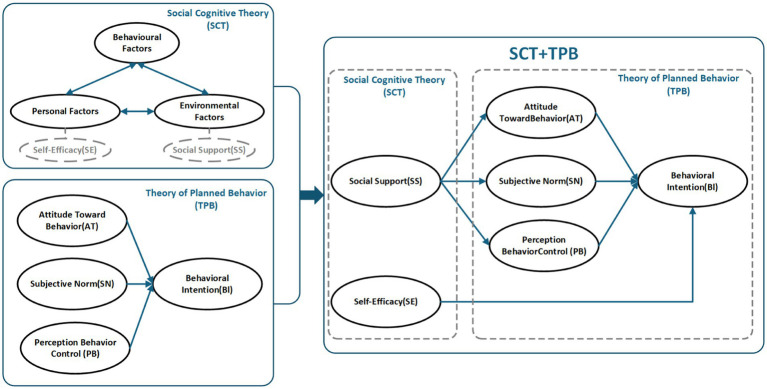
Integrated mechanism model of SCT and TPB.

Social cognitive theory (SCT), developed by Albert Bandura, posits that human behavior is the result of reciprocal interactions among personal, behavioral, and environmental factors (Bandura, 2001). Self-efficacy is regarded as a key psychological mechanism that determines whether individuals are able to initiate a behavior, while social support, as a critical external environmental factor, can shape individuals’ cognitive evaluations of the behavior by providing emotional support, informational resources, and practical conditions. The core mechanisms of SCT are illustrated in Figure 1. The theory of planned behavior (TPB), proposed by Icek Ajzen in 1985, is a psychological model designed to predict and explain deliberate human behavior (Ajzen, 1985; Ajzen, 1991). Its core constructs—attitude toward the behavior, subjective norms, and perceived behavioral control—jointly influence behavioral intention and actual behavior. Its theoretical structure is shown in Figure 1.

While SCT emphasizes the roles of social support and self-efficacy in behavioral formation, TPB asserts that behavioral intention is determined by attitude, subjective norms, and perceived behavioral control. Relying solely on TPB to examine college students’ rural cultural preservation intentions may overlook external social influences, which can be better accounted for through the SCT framework. Conversely, examining behavioral intention solely from the perspective of SCT may neglect individual internal drivers. In this study, social support (SS) is conceptualized as an external social-contextual factor within social cognitive theory (SCT), rather than as a mediating variable. As an external factor, SS indirectly influences behavioral intention by affecting internal psychological factors such as attitude toward the behavior (AT), subjective norms (SN), and perceived behavioral control (PB). Meanwhile, self-efficacy, a core component of SCT, can directly affect an individual’s behavioral intention, thereby influencing actual behavior. While the theory of planned behavior (TPB) emphasizes internal determinants of behavioral intention, SCT focuses on external determinants. The integration of SCT and TPB provides a comprehensive framework for understanding university students’ engagement in rural cultural preservation, as it accounts for both individual internal factors and the influence of social and environmental contexts on behavior. Accordingly, this study structurally integrates SCT and TPB to construct a more comprehensive model of students’ rural cultural preservation behavior from the perspective of the “external environment → internal cognition → behavioral intention” pathway. The integrated mechanism model is illustrated in Figure 1.

### 2.2 Research hypotheses

#### 2.2.1 Influence of attitude toward the behavior on behavioral intention

Attitude toward the behavior refers to an individual’s evaluation of the degree of favorability or unfavorability toward performing a specific behavior, reflecting salient beliefs about its outcomes (Fishbein and Ajzen, 1975). It is regarded as a key predictor of individual behavioral decision-making. Empirical studies in diverse domains—such as help-seeking behaviors among nursing students (Alqhtani et al., 2025) and intentions to implement STEAM education among math teachers—have consistently validated attitude as a critical determinant of behavioral intention (Zhang et al., 2022; Drueke et al., 2021; Smeda et al., 2018).

*H1*: Attitude toward the behavior significantly influences college students’ intention to engage in rural cultural preservation.

#### 2.2.2 Influence of subjective norm on behavioral intention

Subjective norm refers to the perceived social pressure an individual feels regarding whether or not to perform a particular behavior. It plays a significant role in shaping behavioral decisions. Prior research has shown that subjective norms positively influence residents’ donation intentions (Zhao et al., 2025) and managers’ behavioral intentions regarding AI adoption (Pozzo et al., 2024). When individuals perceive social expectations, peer endorsement, or organizational pressure, they are more likely to form a positive behavioral intention (Wijaya and Weinhandl, 2022).

*H2*: Subjective norm positively influences college students’ intention to engage in rural cultural preservation.

#### 2.2.3 Influence of perceived behavioral control on behavioral intention

Perceived behavioral control is defined as an individual’s perception of their ability to perform a given behavior. Studies have demonstrated its positive effect on students’ intention to use online learning tools (Wang et al., 2024), Turkish teachers’ adoption of educational technologies (Kilinc et al., 2016), and college students’ registration for MOOCs (Wang, 2023). These findings consistently show that perceived behavioral control significantly shapes behavioral intention (Shen, 2023; Courtois et al., 2014).

*H3*: Perceived behavioral control significantly influences college students’ intention to engage in rural cultural preservation.

#### 2.2.4 Influence of social support on attitude

Social support refers to the emotional and material assistance an individual receives through social relationships. As a crucial environmental factor, social support can influence behavioral attitude by shaping cognitive and emotional states. For example, studies on the impact of social networks on subjective well-being have found that social support enhances positive behavioral attitudes and self-efficacy, thereby increasing subjective well-being (Cai et al., 2025). Similarly, research on smoking cessation interventions suggests that social support improves behavioral persistence through attitude enhancement (Orouji et al., 2017).

*H4*: Social support positively influences college students’ behavioral attitudes, thereby indirectly affecting their intention to engage in rural cultural preservation.

#### 2.2.5 Influence of social support on subjective norm

Social support strengthens subjective norms, thereby influencing behavioral attitudes and actual behavior. Research on stem cell transplant survivors found that social support reinforced social expectations and group influence, improving medical adherence and reducing psychological distress (Nørskov et al., 2021). Social support has thus been shown to shape subjective norms and influence behavioral intention by mitigating stress and enhancing self-efficacy.

*H5*: Social support positively influences college students’ subjective norms, indirectly affecting their intention to engage in rural cultural preservation.

#### 2.2.6 Influence of social support on perceived behavioral control

Social support enhances perceived behavioral control, thereby improving one’s ability and willingness to act. Studies in health behavior contexts found that social support significantly increased the frequency of healthy behaviors by enhancing perceived control (Hinz et al., 2025). Research on alcohol use reduction also showed that support networks boost behavioral sustainability by strengthening perceived control (Moon et al., 2019). Emotional, informational, and practical support enhances individuals’ sense of control, reinforcing their intention to act.

*H6*: Social support positively influences college students’ perceived behavioral control, thereby indirectly affecting their intention to engage in rural cultural preservation.

#### 2.2.7 Influence of self-efficacy on behavioral intention

Self-efficacy is defined as an individual’s belief in their capability to successfully perform a specific task or behavior (Yang and Delgado, 2025). In experimental studies on compensation settings, general self-efficacy was positively associated with individual willingness to exert effort (Zhang et al., 2015). Research on elementary school students’ math learning also showed that enhancing self-efficacy led to higher learning motivation. These findings confirm that self-efficacy plays a significant role in shaping behavioral intention.

*H7*: Self-efficacy positively influences college students’ intention to engage in rural cultural preservation.

### 2.3 Research model

**Figure 2:**
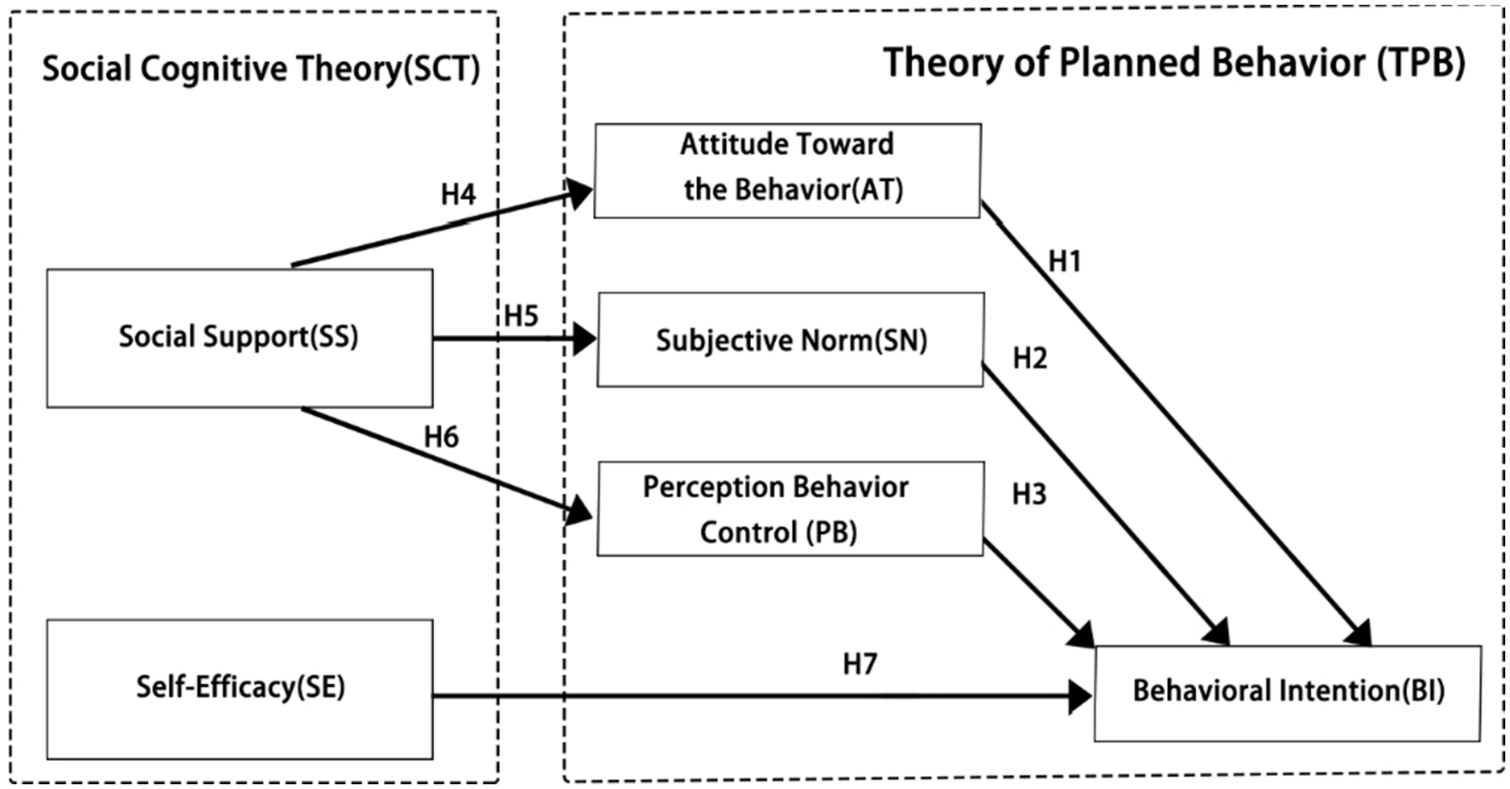
Proposed research model.

Based on a comprehensive review of the relevant literature, this study proposes seven hypotheses and develops a conceptual model to examine college students’ intentions toward rural cultural preservation in the context of rural revitalization. The model incorporates the frameworks of the theory of planned behavior (TPB) and social cognitive theory (SCT) to systematically explain the underlying factors influencing students’ intention to engage in rural cultural preservation. The structural components of the model are visually illustrated in Figure 2.

## 3 Methods

### 3.1 Research process steps

Data for this study were collected through a combination of online and offline questionnaire surveys. The online survey was administered using the Wenjuanxing platform, while the offline component involved paper-based questionnaires completed on site. The purpose was to explore the factors influencing college students’ intention to engage in rural cultural preservation.

**Figure 3:**
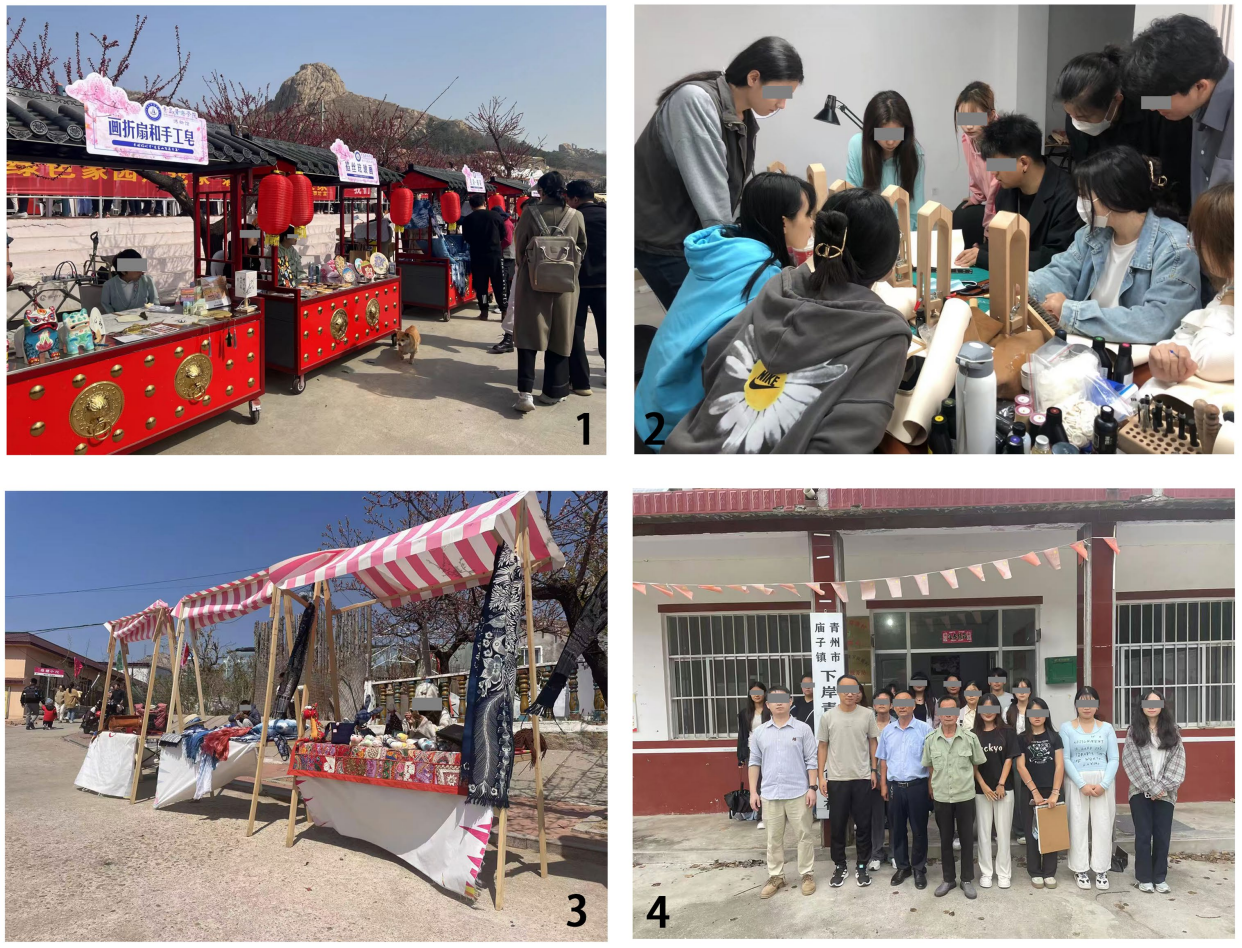
College students’ participation in rural cultural preservation activities. (1) Photographed in 2024 at the Feihua Market, Maojiashan, Lingshanwei Subdistrict, Qingdao. (2) Photographed in 2024 during a class on intangible cultural heritage, taught by Professor Wang Kai at Qingdao Huanghai University. (3) Photographed in 2025 at the Feihua Market, Maojiashan, Lingshanwei Subdistrict, Qingdao. (4) Photographed in 2025 during a rural cultural research and fieldwork course conducted by Environmental Design majors.

The survey was conducted at four universities in China (see Figure 3). Participants were college students who had taken part in rural intangible cultural heritage workshops, field courses related to rural culture, or the “Sanxiaxiang” social service program.

This study adopted a mixed-methods approach, integrating both qualitative and quantitative methodologies. The qualitative component focused on exploring the integrated TPB–SCT theoretical framework, while the quantitative component involved empirical analysis using questionnaire data and the SEM–ANN hybrid method.

**Figure 4:**
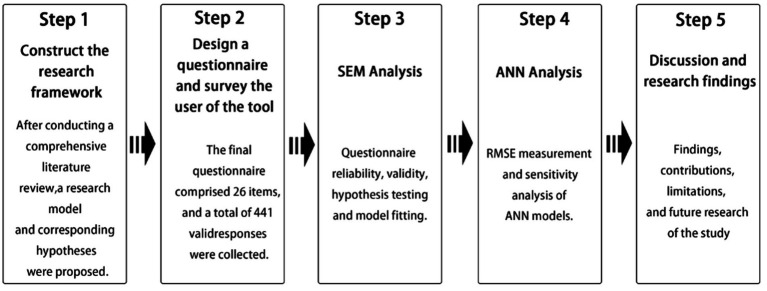
Steps in the research process.

The research was conducted in five main steps: (1) Developing and examining the theoretical framework, including the formulation of the research model and hypotheses; (2) designing a questionnaire based on the proposed hypotheses and administering it to college students who had prior experience with rural cultural activities; (3) conducting structural equation modeling (SEM) to test the proposed hypotheses using the collected survey data; (4) applying artificial neural network (ANN) analysis to assess model stability and validate the robustness of SEM results; (5) interpreting the theoretical and practical implications of the findings (as illustrated in Figure 4).

### 3.2 Questionnaire design

The questionnaire used in this study was developed based on a comprehensive literature review and refined through iterative validation, following established principles and considerations of questionnaire design (Meyer and Henke, 2023). During the development of relevant variables, multiple rounds of expert consultations were conducted to finalize the specific measurement items. After the initial draft was completed, a pilot test was conducted with a group of ten participants. Based on their feedback, modifications were made to the wording and formatting, resulting in the finalized questionnaire.

The questionnaire consisted of two sections. The first section collected demographic information, including gender, age group, education level, and academic major. The second section, which formed the core of the questionnaire, measured students’ attitude toward the behavior, subjective norms, perceived behavioral control, social support, and self-efficacy. Items such as “I believe that rural cultural preservation is meaningful” and “I am eager to participate in rural cultural preservation” were used to examine students’ attitudes, perceived social pressure, and behavioral control, as well as to evaluate the indirect effects of social support on these constructs and the direct effect of self-efficacy on behavioral intention. All items were measured using a five-point Likert scale. As the questionnaire was originally developed in Chinese, a back-translation method was adopted to ensure translation accuracy (see Table 1).

**Table 1:** Behavioral measurement scale.

Variables	Code	Content	Original reference
Attitude toward the behavior (AT)	AT1	I believe that rural cultural preservation is a meaningful endeavor.	Drueke et al. (2021), Rafique et al. (2023), Ragheb and Beard (1982), and Sparks and Pan (2009)
	AT2	I am very willing to participate in rural cultural preservation.	
	AT3	I enjoy rural culture and am willing to participate in its preservation.	
	AT4	Participating in rural cultural preservation helps me broaden my horizons.	
Subjective norm (SN)	SN1	I feel that participating in rural cultural preservation is generally recognized and encouraged by society.	Chen et al. (2022), Burns et al. (2018), and Lin et al. (2012)
	SN2	I am likely to participate in rural cultural preservation if it is approved by my family.	
	SN3	I am willing to participate in rural cultural preservation if it is recommended or arranged by my school or teachers.	
Perceived behavioral control (PB)	PB1	I believe that I have enough time to engage in rural cultural preservation.	Shen (2023), Liao et al. (2022), Ajzen and Madden (1986), and Han et al. (2010)
	PB2	I believe that I can control whether I participate in rural cultural preservation.	
	PB3	I believe I can continue participating in rural cultural preservation activities despite difficulties.	
	PB4	I believe that the resources and conditions needed to participate in rural cultural preservation are available to me.	
Social support (SS)	SS1	My family encourages me to participate in rural cultural preservation and believes that it is a meaningful activity.	Akhtar et al. (2010) and Li and Yin (2015)
	SS2	My friends or classmates believe that preserving rural culture is a social responsibility that college students should undertake, and they support my participation.	
	SS3	Government platforms and resources, along with frequent public advocacy for the importance of rural cultural preservation, have made it easier for me to participate in such activities.	
	SS4	The university has provided courses, lectures, and practical opportunities related to rural cultural preservation, which have enhanced my interest and capacity to engage in such efforts.	
Self-efficacy (SE)	SE1	I believe I am capable of fully dedicating myself to rural cultural preservation.	Terry and O'Leary (1995)
	SE2	I believe I am capable of successfully carrying out rural cultural preservation.	
	SE3	Participating in rural cultural preservation is perceived as easy for me.	
	SE4	I believe I have sufficient ability to perform rural cultural preservation effectively.	
Behavioral intention (BI)	BI1	I intend to continue engaging in rural cultural preservation.	Alzubaidi et al. (2021)
	BI2	In the future, I intend to engage in work related to rural cultural preservation.	
	BI3	I intend to encourage more people to participate in rural cultural preservation.	

### 3.3 Technical analysis

SPSS 27.0 and AMOS 28.0 were employed for data analysis, in conjunction with the artificial neural network (ANN) approach to enhance the model’s explanatory and predictive power. Initially, SPSS was used to clean the dataset and test for normality by calculating the mean, standard deviation, skewness, and kurtosis, ensuring compliance with the assumptions of normal distribution. Subsequently, both confirmatory factor analysis (CFA) and exploratory factor analysis (EFA) were conducted to evaluate the structural and convergent validity of the scales, while Cronbach’s alpha coefficients were calculated to assess internal consistency.

A structural equation model (SEM) was constructed using AMOS to examine the path relationships among variables, and the statistical significance of the hypothesized paths was evaluated using Estimate, standard error (S.E.), critical ratio (C.R.), and *p*-values. To identify potential nonlinear relationships and interaction effects, a multilayer perceptron (MLP) model in SPSS was utilized, with SEM-significant variables entered as the input layer and outcome variables, including self-efficacy, as the output layer. Ten-fold cross-validation was performed to prevent overfitting, and all variables were standardized to enhance training efficiency.

Normalized importance analysis was conducted to quantify the relative influence of each input variable on behavioral intention, thereby facilitating the precise identification of key predictors. The integration of SEM and ANN effectively overcame the limitations of traditional linear modeling in capturing complex behavioral mechanisms, providing more robust technical support for future theoretical modeling and policy implementation.

### 3.4 Data collection

Data for this study were collected using both the online platform Wenjuanxing and offline paper-based questionnaires. To ensure objectivity and representativeness, a purposive sampling method was employed. Students from four Chinese universities, spanning six academic majors, were selected to represent a diverse and comprehensive sample reflecting college students’ willingness to participate in rural cultural preservation. It should be noted that the sample for this study was primarily drawn from students at four universities in eastern China who participated in rural culture-related courses, social practices, or rural cultural experience activities. The sample was relatively concentrated in terms of academic disciplines, and therefore has certain limitations regarding geographic and disciplinary diversity. Participants were instructed to reflect for 3–5 min before completing either the online or offline questionnaire. Questionnaires submitted in less than 30 s were regarded as carelessly completed and were therefore excluded. Additionally, responses that were identical throughout were removed. A total of 546 students voluntarily and anonymously participated in the survey, with 220 responses collected online and 326 collected offline. After a rigorous screening process, 441 valid responses were retained, resulting in an effective response rate of 80.77%, which aligns with Dillman’s (2007) recommendation. This high participation rate reflects the respondents’ engagement with the topic and ensures the representativeness and reliability of the collected data for further analysis.

This study strictly adhered to ethical standards. Prior to participation, all respondents were fully informed about the purpose of the study and their rights, including the right to withdraw at any point. All data collected were anonymized to maximize the protection of participants’ privacy and maintain strict data confidentiality.

As shown in Table 2, the demographic profile of the respondents was summarized. Among the design-major students, 29.5% (*n* = 130) were male and 70.5% (*n* = 311) were female. The proportion of female participants is relatively high because the survey targeted design-related majors in universities, and in China, female students account for a larger share in art and design programs, as reflected in publicly available data from various universities. Therefore, this gender distribution is consistent with the actual educational context on which the study is based and does not represent a sampling bias. A vast majority of the participants (99.3%, *n* = 438) were between 18 and 30 years old, while only 0.7% (*n* = 3) were under 18. Regarding educational background, 95.2% (*n* = 420) were undergraduate students, and 4.8% (*n* = 21) held associate degrees. The largest academic groups were Environmental Design (42.0%) and Visual Communication Design (19.0%), with smaller representations from Digital Media Design, Painting, Calligraphy, and Applied Arts. Overall, the sample comprised undergraduate and associate degree students, who represent the main demographic of university students in China, thereby providing comprehensive and representative data support for this research.

**Table 2:** Demographic characteristics of respondents.

Variable	Category	Frequency (*n*)	Percentage (%)
Gender	Male	130	29.5%
	Female	311	70.5%
Age	Under 18	3	0.7%
	18–30	438	99.3%
Education	Associate degree	21	4.8%
	Bachelor’s degree	420	95.2%
Major	Environmental design	185	42.0%
	Visual communication design	84	19.0%
	Digital media design	51	11.6%
	Painting	46	10.4%
	Calligraphy	71	16.1%
	Applied arts	4	0.9%

## 4 Data analysis

### 4.1 Descriptive statistics and normality test

Descriptive statistics and normality tests were conducted as shown in Table 3. The skewness values ranged from −0.006 to 0.015, and kurtosis values ranged from −1.219 to −0.837. The means (M) ranged from 3.09 to 3.38, while the standard deviations (SD) ranged from 1.11 to 1.231. Data were considered to exhibit normality when kurtosis and skewness values were within ±10 and ±3, respectively (Moorthy et al., 2019). All available data in this study were considered approximately normally distributed and deemed appropriate for further analysis.

**Table 3:** Results of descriptive statistics analysis and normality test.

Variables	Code	Min	Max	Mean	S.D.	Median	Skewness	Kurtosis
Social support (SS)	SS1	1	5	3.24	1.154	3	−0.132	−0.983
	SS2	1	5	3.15	1.144	3	−0.067	−0.981
	SS3	1	5	3.09	1.201	3	−0.017	−1.066
	SS4	1	5	3.24	1.128	3	−0.164	−0.837
Attitude toward the behavior (AT)	AT1	1	5	3.29	1.185	3	−0.109	−1.121
	AT2	1	5	3.36	1.173	3	−0.086	−1.174
	AT3	1	5	3.35	1.143	3	−0.091	−1.109
	AT4	1	5	3.38	1.122	3	−0.15	−0.924
Subjective norms (SN)	SN1	1	5	3.28	1.231	3	−0.072	−1.212
	SN2	1	5	3.26	1.171	3	−0.006	−1.1
	SN3	1	5	3.33	1.204	3	−0.101	−1.108
Perceived behavioral control (PB)	PB1	1	5	3.34	1.151	3	−0.069	−1.078
	PB2	1	5	3.33	1.123	3	−0.171	−0.982
	PB3	1	5	3.31	1.128	3	−0.041	−1.155
	PB4	1	5	3.34	1.13	3	−0.185	−0.94
Self-efficacy (SE)	SE1	1	5	3.31	1.11	3	−0.085	−0.939
	SE2	1	5	3.27	1.134	3	−0.066	−0.977
	SE3	1	5	3.3	1.15	3	−0.095	−1.093
	SE4	1	5	3.35	1.144	3	−0.032	−1.219
Behavioral intention (BI)	BI1	1	5	3.3	1.142	3	0.015	−1.145
	BI2	1	5	3.28	1.128	3	−0.059	−0.997
	BI3	1	5	3.32	1.171	3	−0.104	−1.06

SS, Social support; AT, Attitude toward the behavior; SN, Subjective norms; PB, Perceptual behavior; SE, Self-efficacy; BI, Behavioral intention.

### 4.2 Measurement model

**Figure 5:**
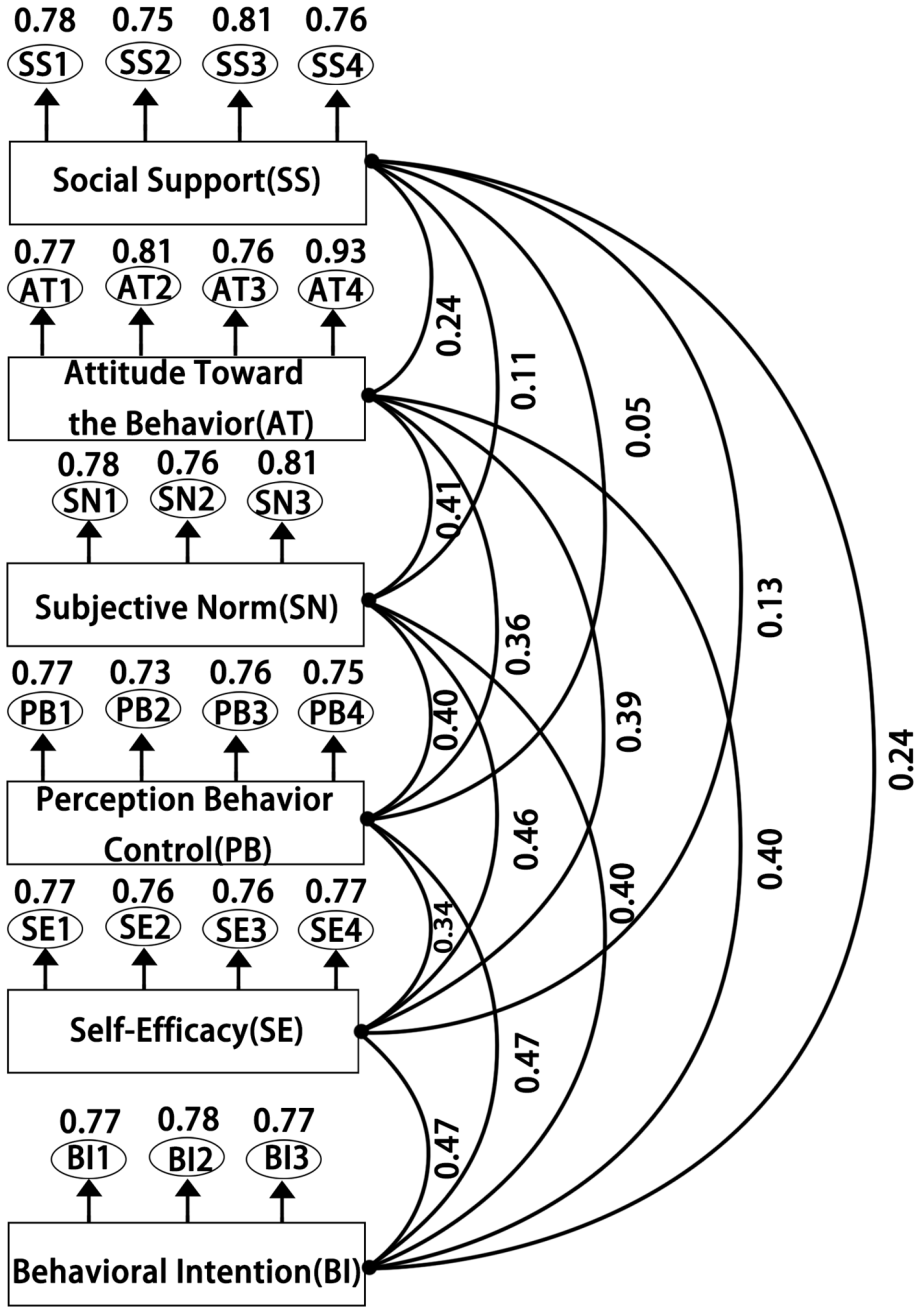
Measurement model.

As shown in Table 4 and Figure 5, the reliability of the measurement model was evaluated using four key indicators: standardized factor loadings (Estimate), Cronbach’s alpha, composite reliability (CR), and average variance extracted (AVE). All standardized factor loadings ranged from 0.725 to 0.926, indicating strong explanatory power across dimensions. Cronbach’s alpha, serving as a reliability metric, demonstrated robust internal consistency, with all values exceeding 0.7 and ranging from 0.817 to 0.883. Composite reliability (CR) was employed to assess the reliability of each construct, with values ranging from 0.818 to 0.888, all above the recommended threshold of 0.7 (Peterson and Kim, 2013). Notably, the attitude dimension achieved the highest CR value of 0.888, further confirming the scale’s internal consistency. All AVE values exceeded the minimum standard of 0.5, with the highest AVE of 0.667 found in the attitude dimension, indicating that 66.7% of the variance was explained by its indicators and reflecting excellent convergent validity. Overall, the results of confirmatory factor analysis (CFA) confirmed the construct validity of the instrument and provided a robust theoretical and methodological basis for subsequent variable measurement. All six dimensions of the scale exhibited high internal consistency, satisfying fundamental psychometric requirements.

**Table 4:** Results of construct validity and reliability analysis.

Construct	Indicator	Estimate	Cronbach’s α	AVE	CR
Social support (SS)	SS1	0.758	0.855	0.597	0.855
	SS2	0.805			
	SS3	0.748			
	SS4	0.778			
Attitude toward the behavior (AT)	AT1	0.765	0.883	0.667	0.888
	AT2	0.808			
	AT3	0.756			
	AT4	0.926			
Perceived behavioral control (PB)	PB1	0.752	0.838	0.565	0.839
	PB2	0.756			
	PB3	0.725			
	PB4	0.773			
Subjective norms (SN)	SN1	0.776	0.823	0.609	0.823
	SN2	0.758			
	SN3	0.806			
Behavioral intention (BI)	BI1	0.775	0.817	0.599	0.818
	BI2	0.781			
	BI3	0.766			
Self-efficacy (SE)	SE1	0.772	0.849	0.584	0.849
	SE2	0.760			
	SE3	0.756			
	SE4	0.769			

α, Cronbach’s alpha; CR, composite reliability; AVE, average variance extracted.

The discriminant validity of the research model refers to whether the indicators measuring a particular construct are weakly correlated with those measuring different constructs. Discriminant validity is considered adequate when the square root of a construct’s average variance extracted (AVE) exceeds its correlations with other constructs (Zhao et al., 2022). As shown in Table 5, significant yet varying degrees of correlations were observed among all psychological constructs, indicating a theoretically sound pattern of relationships. Table 6 shows that all HTMT values are below the 0.85 threshold, indicating good discriminant validity among the constructs.

**Table 5:** Results of discriminate validity of the research model.

Dimension	Social Support (SS)	Attitude Toward the Behavior (AT)	Subjective Norm (SN)	Perceived Behavioral Control (PB)	Self-Efficacy (SE)	Behavioral Intention (BI)
Social support (SS)	0.773					
Attitude toward the behavior (AT)	0.243**	0.817				
Subjective norm (SN)	0.113*	0.407**	0.780			
Perceived behavioral control (PB)	0.046	0.361**	0.396**	0.752		
Self-efficacy (SE)	0.135*	0.390**	0.455**	0.339**	0.764	
Behavioral intention (BI)	0.245**	0.400**	0.397**	0.469**	0.470**	0.774

**p < 0.01, *p < 0.05.

**Table 6:** HTMT (heterotrait-monotrait ratio of correlations) values.

Construct	SS	AT	PB	SN	BI	SE
SS						
AT	0.224					
PB	0.043	0.318				
SN	0.095	0.369	0.329			
BI	0.204	0.363	0.391	0.324		
SE	0.111	0.354	0.287	0.381	0.393	

### 4.3 Structural model

The results indicated that both the measurement and structural models exhibited good model fit, and all indices met the recommended fit criteria. As shown in Table 7, the overall fit of the theoretical model ranged from acceptable to excellent. The model evaluation was conducted in accordance with the recommendations of Hair et al. (2012) and the fit criteria proposed by Hu and Bentler (1999). The CMIN/DF was 2.686, TLI was 0.914, and CFI was 0.925, indicating a high level of fit. Although the RMSEA was slightly higher than the ideal threshold of 0.05, the value of 0.062 was still well below the cutoff of 0.08, indicating acceptable model error. The NFI and GFI were 0.886 and 0.891, respectively, suggesting reasonable explanatory power. Notably, the RMSEA remained around the ideal value of 0.06, and the strong performance of the CMIN/DF further confirmed the absence of major specification errors. Overall, the model fit indices consistently supported the theoretical framework, providing a robust statistical foundation for hypothesis testing and indicating that the structural equation model adequately explained the observed data.

**Table 7:** Model fit indices.

Model	CMIN/DF	RMSEA	NFI	TLI	GFI	CFI
Results	2.686	0.062	0.886	0.914	0.891	0.925
Judgment criterion	1–3	<0.05	>0.9	>0.9	>0.9	>0.9

All indices meet the recommended model fit criteria.

**Figure 6:**
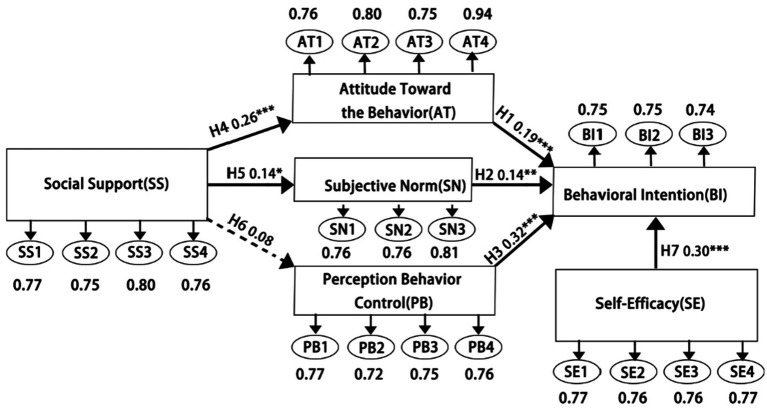
Results of path analysis. All hypotheses are supported, except H6. ***p < 0.001, **p < 0.01, *p < 0.05.

The structural equation modeling (SEM) path analysis results, as presented in Table 8 and Figure 6, revealed the influence mechanisms among psychological constructs and the levels of statistical significance. The direct effect of perceived behavioral control (PB) on behavioral intention (BI) in path H3 was found to be the most significant (*β* = 0.316, *p* < 0.001), followed by the impact of self-efficacy (SE) on BI in path H7 (*β* = 0.299, *p* < 0.001), indicating that perceptions of behavioral difficulty and self-evaluation of capability are the two most critical predictors of behavioral intention.

**Table 8:** Results of path coefficients hypotheses.

Hypotheses	Path	Estimate	S. E.	C. R.	*p*	Result
H1	Attitude Toward the Behavior (AT) → Behavioral Intention (BI)	0.255	0.047	3.624	***	Supported
H2	Subjective Norm (SN) → Behavioral Intention (BI)	0.078	0.047	2.634	0.008**	Supported
H3	Perceived Behavioral Control (PB) → Behavioral Intention (BI)	0.137	0.054	5.576	***	Supported
H4	Social Support (SS) → Attitude Toward the Behavior (AT)	0.143	0.057	4.686	***	Supported
H5	Social Support (SS) → Subjective Norm (SN)	0.299	0.063	2.408	0.016*	Supported
H6	Social Support (SS) → Perceived Behavioral Control (PB)	0.189	0.057	1.382	0.167	Rejected
H7	Self-Efficacy (SE) → Behavioral Intention (BI)	0.316	0.054	5.343	***	Supported

C.R., Critical ratio; S.E., Standardized estimates; SS, Social support; AT, Attitude toward the behavior; SN, Subjective norms; PB, Perceptual behavior; SE, Self-efficacy; BI: Behavioral intention. ***p < 0.001, **p < 0.01, *p < 0.05.

Path H4 was also statistically significant (*β* = 0.255, *p* < 0.001), whereas path H6 failed to reach statistical significance (*β* = 0.078, *p* = 0.167), suggesting that social support (SS) may influence BI indirectly by shaping individual attitudes rather than affecting perceived behavioral control. Notably, all three paths leading to BI (H1: *β* = 0.189, H2: *β* = 0.143, H3: *β* = 0.316) were statistically significant, thereby validating the core assumptions of the Theory of Planned Behavior (TPB).

Additionally, the marginal effect of SS on subjective norms (SN) in path H5 (*β* = 0.137, *p* = 0.016) implies that social environmental factors may affect behavioral decision-making through various complex mechanisms. All significant paths exhibited critical ratio (C.R.) values greater than 2.4, with the path from PB to BI demonstrating the highest C.R. value (5.576), further confirming the robustness of this relationship.

Table 9 reports the indirect effects of social support (SS) on behavioral intention via internal psychological variables. SS significantly influenced behavioral intention through attitude toward the behavior (AT) (Estimate = 0.048, 95% confidence interval of [0.014, 0.097], *p* = 0.004), but its indirect effect via perceived behavioral control (PB) was not significant (Estimate = 0.025, 95% confidence interval of [−0.011, 0.075], *p* = 0.199). The pathway through subjective norms (SN) was significant albeit weaker than AT (Estimate = 0.020, 95% confidence interval of [0.001, 0.058], *p* = 0.036), indicating that SN, as an internal psychological variable, plays a certain mediating role in the effect of SS on behavioral intention. However, its effect is weaker than the pathway through AT.

**Table 9:** Analysis of the effects of social support on behavioral intention.

Mediation pathway	Estimate	Lower	Upper	*p*
Social Support (SS) → Attitude Toward the Behavior (AT) → Behavioral Intention (BI)	0.048	0.014	0.097	0.004
Social Support (SS) → Subjective Norm (SN) → Behavioral Intention (BI)	0.020	0.001	0.058	0.036
Social Support (SS) → Perceived Behavioral Control (PB) → Behavioral Intention (BI)	0.025	−0.011	0.075	0.199

### 4.4 Artificial neural network analysis (ANN)

To minimize potential limitations associated with SEM, an artificial neural network (ANN) analysis was employed to complement the structural equation modeling approach (Tan et al., 2014). ANN is capable of modeling both linear and nonlinear relationships without assuming data normality (Teo et al., 2015). Moreover, ANN is effective in simulating human decision-making processes, independent of covariate-independent variable interactions, and does not require the assumption of linear relationships when predicting outcomes based on multiple inputs (Wilson and Bettis-Outland, 2020).

Given that the SEM has confirmed the significance of the main hypothesized paths, a multilayer perceptron (MLP) artificial neural network (ANN) model was introduced to further explore potential nonlinear relationships among the variables. Based on the SEM results, attitude toward the behavior (AT), subjective norms (SN), perceived behavioral control (PB), and self-efficacy (SE) were included as input nodes in the ANN, while behavioral intention (BI) was set as the output variable. Social support (SS) was excluded from the input layer, as it only exerts indirect effects. The ANN employed a single hidden layer structure (default setting in SPSS MLP), with the number of hidden nodes automatically determined by the software for optimal fit. A Sigmoid activation function was used for the hidden layer, and a linear function was applied to the output layer to accommodate the continuous dependent variable. A ten-fold cross-validation strategy was employed, in which the sample was randomly divided in a 9:1 ratio, with 90% used for model training and 10% for testing.

**Figure 7:**
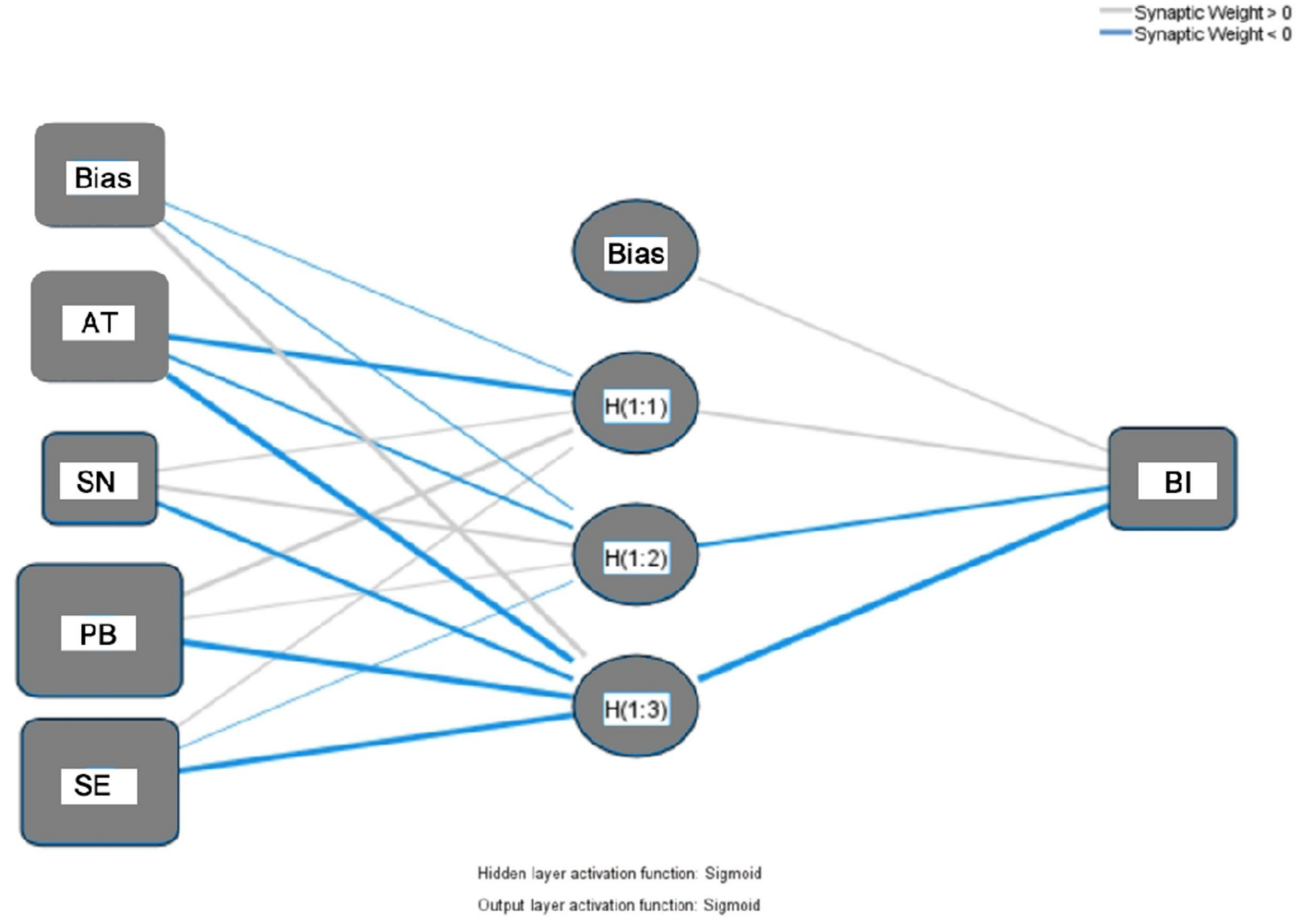
ANN model.

In addition, all input and output variables were normalized to a [0, 1] range prior to model training to enhance efficiency and reduce training time, while improving predictive performance (Liébana-Cabanillas et al., 2018). Ultimately, the constructed ANN model successfully predicted college students’ behavioral intentions toward rural cultural preservation. This result not only reaffirmed the stability of the SEM findings but also revealed deeper nonlinear relationships among the variables, as illustrated in Figure 7.

As noted in the literature, the root mean square error (RMSE) is an indicator used to evaluate the predictive accuracy of a model (Chong, 2013; Leong et al., 2013). The average cross-validation RMSE of the training model was 0.364, while the RMSE of the testing model was 0.355 (see Table 10). These results indicate that the ANN model demonstrates reliability in capturing the numerical relationships between the predictive factors and outcomes.

**Table 10:** RMSE values of artificial neural networks models.

ANN	Training	Testing
ANN1	0.352	0.302
ANN2	0.358	0.266
ANN3	0.430	0.345
ANN4	0.364	0.395
ANN5	0.369	0.396
ANN6	0.370	0.399
ANN7	0.338	0.354
ANN8	0.347	0.441
ANN9	0.346	0.367
ANN10	0.366	0.281
Mean	0.364	0.355
SD	0.160	0.238

In the artificial neural network (ANN) model constructed in this study to predict college students’ intentions toward rural cultural preservation behavior, sensitivity analysis was employed to quantify the relative influence of each independent variable on the output variable. Sensitivity analysis primarily evaluates the “importance” of each variable by assessing the extent to which changes in that variable influence the ANN output (Chan and Chong, 2012); while normalized importance reflects the relative contribution of each variable as a percentage of the most influential variable, which is set at 100%. As shown in Table 10, the most influential predictors in the ANN model for forecasting college students’ intentions to engage in rural cultural preservation were attitude toward the behavior (AT), subjective norm (SN), perceived behavioral control (PB), and self-efficacy (SE).

Based on the SEM results confirming several hypotheses, attitude toward the behavior (AT), perceived behavioral control (PB), subjective norm (SN), and self-efficacy (SE) were selected as predictor variables in the artificial neural network (ANN) model, while social support (SS) was excluded due to its indirect effect on Behavioral Intention (BI). A ten-fold cross-validation was performed to avoid model overfitting, with 90% of the data used for training and 10% for testing.

In the model with BI as the output variable, the predictor rankings were completely consistent between the SEM and ANN approaches, indicating a high level of agreement. PB ranked first (*β* = 0.316; NI = 100.00%), followed by SE (*β* = 0.299; NI = 86.086%). These findings confirm, from multiple statistical perspectives, that PB and SE are the most critical predictors of college students’ intentions to engage in rural cultural preservation. This path showed strong consistency across both modeling methods.

The final ANN model, detailed in Table 11, was used to assess Behavioral Intention, verifying the robustness of the SEM findings and enabling deterministic prediction. The root mean square error (RMSE) was employed as an indicator of predictive accuracy (Chong, 2013).

**Table 11:** Neural network sensitivity analysis.

ANN	AT	SN	PB	SE
ANN1	0.196	0.175	0.356	0.273
ANN2	0.212	0.123	0.349	0.316
ANN3	0.134	0.360	0.444	0.062
ANN4	0.217	0.092	0.375	0.316
ANN5	0.112	0.170	0.423	0.295
ANN6	0.189	0.136	0.317	0.358
ANN7	0.243	0.161	0.290	0.306
ANN8	0.167	0.163	0.295	0.375
ANN9	0.228	0.180	0.294	0.298
ANN10	0.267	0.056	0.305	0.371
Average imporance	0.197	0.162	0.345	0.297
Normanlized importance (%)	57.101	46.957	100.000	86.086

Overall, the cross-validation between SEM and ANN not only enhanced the theoretical robustness of the model’s key paths but also compensated for the structural equation modeling’s limitations in capturing higher-order variable influences and path heterogeneity through the ANN’s capacity to detect nonlinear interactions among variables. This integrated approach effectively revealed the more complex psychological structures and influencing mechanisms underlying students’ behavioral intentions, providing multidimensional empirical support for understanding college students’ decision-making in rural cultural preservation efforts (see Table 12).

**Table 12:** SEM-ANN comparison results analysis.

Model 1 (output: BI)	SEM: path coefficient	ANN: normanlized importance (%)	SEM ranking	ANN ranking	Remark
AT	0.189	57.101	3	3	Match
SN	0.143	46.957	4	4	Match
PB	0.316	100.000	1	1	Match
SE	0.299	86.086	2	2	Match

## 5 Discussion

This study employed a SEM-ANN empirical approach to investigate the factors influencing college students’ behavior in rural cultural preservation within the broader framework of rural revitalization. In the SEM phase, six out of the seven proposed hypotheses were confirmed as statistically significant, while one was not. In the ANN phase, sensitivity analysis was conducted to determine the importance of predictive factors, with the results in Tables 11, 12 demonstrating consistency between the two analytical stages. The following sections summarize and discuss the study’s findings.

### 5.1 Discussion of supported hypotheses

First, consistent with previous findings, H1 (*β* = 0.189, *p* < 0.001), H2 (*β* = 0.143, *p* = 0.008), and H3 (*β* = 0.316, *p* < 0.001) were empirically supported, validating the core constructs of the theory of planned behavior (TPB)—Attitude toward the behavior (AT), perceived behavioral control (PB), and subjective norms (SN)—as joint determinants of behavioral intention (BI) and actual behavior (Ajzen, 1985). Among them, PB exerted the most significant impact on BI (H3, *β* = 0.316, *p* < 0.001), suggesting that students’ perceived ability to engage in rural cultural preservation serves as a key driver of behavioral intention. This finding is consistent with the foundational assumptions of TPB and implies that enhancing students’ confidence and perceived competence is central to motivating actual engagement. Additionally, AT (H1, *β* = 0.189, *p* < 0.001) had a significant positive influence on BI. These results indicate that when the importance and value of rural cultural preservation are recognized by students, and when positive emotions are held toward the activities, their willingness to participate is significantly strengthened. Therefore, the cultivation of cultural identity, a sense of responsibility, and emotional resonance should be prioritized as a strategic entry point for behavioral activation. Although the path coefficient of SN (H2, *β* = 0.143, *p* = 0.008) was relatively lower, statistical significance was still achieved, indicating that expectations and support from family, institutions, and society contribute to enhancing BI. Nevertheless, the effect appears to be relatively modest compared to individual cognitive evaluations, suggesting a high degree of autonomy and independence among contemporary students in their value-based decisions.

Second, within the framework of social cognitive theory (SCT), self-efficacy (SE) was found to have a significant influence on BI (H7, *β* = 0.299, *p* < 0.001), with a normalized importance score of 86.086% in the ANN model—second only to PB. This highlights the critical psychological mechanism through which individuals’ belief in their ability to perform a given behavior shapes intention. Practical strategies such as field visits, task decomposition, and feedback-based achievement reinforcement may be highly effective in strengthening students’ perceived competence and willingness to act.

Third, although social support (SS) exerted significant indirect effects on behavioral intention (BI) via attitude toward the behavior (AT) (H4, *β* = 0.255, *p* < 0.001) and subjective norms (SN) (H5, *β* = 0.137, *p* = 0.016), it was not treated as a mediating variable in this study, but rather as an external social-contextual factor. The significant indirect effects of SS on BI follow the pathway logic of “external factor → internal psychological variable → behavioral intention,” which is consistent with the model proposed in this study. As an external social factor, SS can play a positive role in cognitive construction and the formation of attitudes. In particular, the indirect effect through the path SS → AT → BI (Estimate = 0.048, *p* = 0.004) was found to be stronger than alternative pathways, suggesting that SS primarily facilitates behavioral intention by shaping positive attitudes rather than by directly enhancing perceived control. This finding offers practical implications for behavior-oriented interventions: social support efforts should emphasize fostering a sense of identity and moral responsibility, beyond merely providing external encouragement.

Fourth, in this study, perceived behavioral control (PB) and self-efficacy (SE) emerged as the strongest predictors, which can be explained from the perspective of motivation theory. According to Expectancy–Value Theory, judgments of capability are central to the formation of behavioral intention; thus, when university students perceive that they possess the ability and resources to engage in rural cultural preservation, their intention to act naturally increases. Self-Determination Theory also emphasizes that competence is a key driver of intrinsic motivation, and higher self-efficacy is associated with a greater likelihood of forming stable behavioral tendencies. Additionally, PB, as a direct source of perceived behavioral feasibility, enables individuals to view the behavior as “performable and controllable,” thereby reducing psychological resistance. Overall, PB and SE together constitute an ability-based motivational mechanism: PB emphasizes external feasibility, SE reinforces internal capability beliefs, and when both are strong, they substantially enhance students’ intention to participate in rural cultural preservation.

In sum, the integration of TPB and SCT constructs was validated as an effective explanatory framework for college students’ intention to engage in rural cultural preservation. Notably, the consistent ranking of PB, SE, and AT across both SEM and ANN analyses reinforces the stability and predictive power of these variables. Future interventions should therefore focus on enhancing students’ sense of behavioral control, self-efficacy, and attitudinal identification, supplemented by positive environmental guidance to form a synergistic mechanism combining internal motivation and external support.

### 5.2 Discussion of non-significant hypothesis

First, among all the tested hypotheses, only the H6 path—suggesting a positive impact of social support (SS) on perceived behavioral control (PB)—was not found to be statistically significant (*β* = 0.078, *p* = 0.167). This suggests that, within the context of this study, social support did not significantly enhance students’ perceptions of control over engaging in rural cultural preservation behaviors. Although social support has generally been considered to enhance individuals’ confidence and perceived control in behavioral contexts, the findings indicate that students’ evaluations of the ease or difficulty of participating in such activities were more dependent on personal experiences, perceived competencies, and real-world feasibility factors (e.g., time availability, knowledge preparation), rather than on the availability of external support. In other words, although emotional and informational encouragement was provided by institutions such as government agencies, universities, families, and peers, such support was insufficient to shift students’ subjective assessments of whether the behavior could be successfully carried out.

Second, based on the structural model and mediation analysis, social support (SS), as an external social-contextual factor, exerted significant indirect effects on behavioral intention (BI) through attitude toward the behavior (AT) and subjective norms (SN), whereas the pathway via perceived behavioral control (PB) was not significant. This finding implies that social support may be more effective in shaping value recognition and perceived social norms than in directly improving students’ perceived behavioral competence. Such a result is consistent with prior studies, which have shown that when support remains at the level of macro-level advocacy or emotional encouragement, its ability to enhance concrete behavioral execution is limited (Moon et al., 2019; Hinz et al., 2025). Therefore, in order to effectively strengthen students’ perceptions of behavioral control regarding rural cultural preservation, future policy or programmatic interventions should prioritize the allocation of practical resources—such as skill training, guided action frameworks, task decomposition, and structured feedback mechanisms—rather than merely focusing on promotional messaging or ideological appeal.

This finding provides critical insight for universities and policymakers in designing more actionable strategies to promote student engagement in rural revitalization efforts. Beyond amplifying external incentives, interventions should be grounded in tangible engagement mechanisms that can strengthen students’ awareness of their own capabilities, thereby facilitating the formation and transformation of behavioral intention into actual participation.

### 5.3 Discussion of linear and non-linear validation

This study was conducted using a two-stage analytical approach, which integrated structural equation modeling (SEM) and artificial neural networks (ANN), to systematically identify the key determinants of college students’ intentions to engage in rural cultural preservation. During the linear validation stage, the SEM model—grounded in the SCT and TPB frameworks—confirmed that perceived behavioral control (PB), self-efficacy (SE), attitude toward the behavior (AT), and subjective norms (SN) exerted significant positive effects on behavioral intention (BI). Among them, PB (*β* = 0.316) and SE (*β* = 0.299) emerged as the most influential predictors, highlighting the importance of perceived controllability and confidence in one’s abilities as central drivers of behavioral intention.

To additionally explore the potential nonlinear relationships and higher-order interactions among variables, the ANN model was employed. In the ANN sensitivity analysis, the four primary predictors were ranked in descending order of normalized importance as follows: PB (100.000%) > SE (86.086%) > AT (57.101%) > SN (46.957%). This ranking was highly consistent with the SEM findings, which reinforces the robustness and predictive accuracy of the combined model. Since ANN does not require assumptions of data normality or variable independence, it was particularly suitable for detecting nonlinear patterns and interaction effects—especially the synergistic or diminishing marginal effects often observed in psychological variables.

It is noteworthy that although social support (SS) did not significantly influence PB within the SEM model, it exerted indirect effects via AT and SN pathways. Moreover, SS was not included as an input variable in the ANN model, which further substantiates its role as an indirect rather than direct determinant. This layered modeling strategy revealed the mediating role of psychological constructs in linking external social support to internal decision-making, thereby enriching the theoretical explanatory depth.

In summary, the dual methodology of SEM and ANN not only demonstrated a high degree of consistency in model validation and key path identification but also addressed the limitations of traditional SEM in handling asymmetric effects and variable interactions. This integrative framework offers a more multidimensional and comprehensive understanding of behavioral intention formation among university students. As such, it provides a pragmatic modeling paradigm and empirical reference for future studies in behavioral science, educational interventions, and policy evaluation.

## 6 Conclusion

### 6.1 Theoretical contributions

Significant theoretical advancements were achieved in this study, primarily through the integration of complementary behavioral theories. A more comprehensive explanatory model was developed through the first systematic integration of the theory of planned behavior (TPB) and social cognitive theory (SCT), effectively addressing the limitations posed by relying on a single theoretical framework in prior research. Whereas TPB focuses on internal psychological constructs such as attitude toward the behavior, subjective norms, and perceived behavioral control, SCT emphasizes external social influences such as social support and self-efficacy. The integration of these two perspectives provides a more holistic understanding of the formation mechanism underlying university students’ intentions to participate in rural cultural preservation. This integration not only enhanced the applicability of the theoretical framework but also facilitated the interdisciplinary application of both theories within the context of rural culture research, thereby laying a solid theoretical foundation for future investigations.

Methodologically, an innovative two-stage analytical approach combining structural equation modeling (SEM) and artificial neural networks (ANN) was introduced, overcoming the limitations of traditional linear modeling in capturing complex variable relationships. While SEM was used to verify the path relationships and assess model fit, ANN was employed to identify nonlinear associations and determine variable importance rankings, thereby advancing from theoretical validation to predictive modeling. This multi-model collaborative paradigm enhanced the robustness and explanatory power of the findings and offered a novel methodological path for exploring complex behavioral mechanisms in the social sciences. Moreover, by focusing on university students as the research population, the study addressed the practical need to examine youth engagement under the rural revitalization strategy, filling a theoretical gap in the literature on rural cultural preservation within higher education contexts.

### 6.2 Practical implications

Practical intervention strategies and operational recommendations were provided for universities, governments, and civil society to enhance university students’ engagement in rural cultural preservation. Perceived behavioral control (PB) and self-efficacy (SE) were identified as the most influential predictors of behavioral intention (BI), highlighting the importance of improving students’ self-perceived abilities and their perceived feasibility of the behavior in fostering motivation to participate. Universities are encouraged to strengthen students’ competencies through curriculum development, field research, and hands-on projects, thereby enhancing their understanding and confidence in undertaking rural cultural preservation efforts.

Meanwhile, the study also found that social support (SS), as an external social-contextual variable, indirectly influenced behavioral intention (BI) through attitude toward the behavior (AT) and subjective norms (SN), highlighting the importance of fostering a positive social environment and guiding public opinion. Cultural identity can be reinforced through the integration of ideological education, thematic campaigns, and the promotion of exemplary role models in academic settings. In addition, sustainable support mechanisms and experiential platforms, such as “Intangible Cultural Heritage Workshops” and “Rural Revitalization Camps,” should be provided by governments and communities to strengthen students’ sense of social responsibility and belonging.

In summary, this study not only provides a theoretical foundation for understanding university students’ behaviors related to rural cultural preservation but also offers practical guidance for educational institutions, cultural agencies, and policymakers involved in rural revitalization initiatives.

### 6.3 Limitations and future research

Despite the exploratory efforts made in theoretical integration and methodological application, several limitations should be acknowledged and addressed in future research. First, the data were primarily collected from students at four universities located in eastern China. Although the sample holds a certain degree of representativeness, limitations persist in terms of regional, cultural, and disciplinary diversity, which may constrain the external generalizability of the findings. Future studies should adopt large-scale surveys across multiple regions and institutions to enhance the generalizability and robustness of the results.

Second, a cross-sectional data collection approach was employed, which only captured students’ behavioral intentions at a single point in time. Consequently, this approach was insufficient for uncovering dynamic behavioral changes or long-term developmental trends. Longitudinal tracking designs should be implemented in subsequent studies to examine how students’ behavioral intentions evolve across different stages of rural cultural protection engagement.

Third, while the dual-model framework integrating SEM and ANN effectively identified key causal paths and variable importance, it did not explore potential moderating or mediating mechanisms. Future research should incorporate multi-group comparisons, hierarchical linear modeling, or moderated structural equation modeling to assess how demographic or experiential factors (e.g., gender, academic background, rural familiarity) may influence behavioral intention pathways.

Finally, this study focused solely on behavioral intention without empirically validating the transition to actual behavior. Future investigations should target participants with practical experience in rural cultural preservation, such as student volunteers, and employ qualitative interviews or case-based approaches to explore the pathways through which behavioral intention transforms into real-world actions. This would enhance the applicability of research outcomes in policymaking and educational practice.

## Data Availability

The original contributions presented in the study are included in the article/supplementary material, further inquiries can be directed to the corresponding author.
